# Surface Modification Progress for PLGA-Based Cell Scaffolds

**DOI:** 10.3390/polym16010165

**Published:** 2024-01-04

**Authors:** Bohua Yan, Yabing Hua, Jinyue Wang, Tianjiao Shao, Shan Wang, Xiang Gao, Jing Gao

**Affiliations:** 1 State Key Laboratory of Toxicology and Medical Countermeasures, Beijing Institute of Pharmacology and Toxicology, Beijing 100850, China; super_yanbohua@163.com (B.Y.); 18733347430@163.com (J.W.); tianjiao0060@163.com (T.S.); wsss980503@163.com (S.W.); 2 Department of Pharmacy, Xuzhou Medical University Jiangsu Key Laboratory of New Drug Research and Clinical Pharmacy, Xuzhou Medical University, Xuzhou 221004, China; huayabing@xzhmu.edu.cn

**Keywords:** PLGA, surface modification, cell scaffold, cell delivery, cell adhesion

## Abstract

Poly(lactic-glycolic acid) (PLGA) is a biocompatible bio-scaffold material, but its own hydrophobic and electrically neutral surface limits its application as a cell scaffold. Polymer materials, mimics ECM materials, and organic material have often been used as coating materials for PLGA cell scaffolds to improve the poor cell adhesion of PLGA and enhance tissue adaptation. These coating materials can be modified on the PLGA surface via simple physical or chemical methods, and coating multiple materials can simultaneously confer different functions to the PLGA scaffold; not only does this ensure stronger cell adhesion but it also modulates cell behavior and function. This approach to coating could facilitate the production of more PLGA-based cell scaffolds. This review focuses on the PLGA surface-modified materials, methods, and applications, and will provide guidance for PLGA surface modification.

## 1. Introduction

In recent years, methods that deliver cells in vivo to repair tissue damage have rapidly developed. The safe and efficient delivery of cells has attracted widespread attention. Studies have shown that transplanted cells had a higher viability when provided with an environment that was similar to that of normal tissue at the site of tissue damage [1]. The extracellular matrix (ECM) contains various active components, such as hyaluronic acid (HA), laminin, fibronectin (FN), and collagen, which regulate the life activities of the cells and enable cell adhesion, growth, proliferation, migration, communication, and other regulatory activities. Among these regulatory activities, cell adhesion motifs such as REDV (Arg-Glu-Asp-Val), RGD (Arg-Gly-Asp), and YIGSR (Tyr-Ile-Gly-Ser-Arg) [2] comprise the material basis of cell adhesion. Tissue engineering scaffolds can provide a 3D space and a large internal volume, which allows cells to attain sufficient nutrition and enables them to communicate with each other, and the metabolites can excrete in time. These scaffolds offer a similar environment to ECM for cell growth; however, different types of scaffolds should be selected depending on the tissue damage site and number of defects.

Poly(lactic-glycolic acid) (PLGA) has been approved by the United States Food and Drug Administration (FDA)/European Medicines Agency (EMA) due to its good biocompatibility, degradability, easy processability, and tunable mechanical strength, and it has been widely used in tissue engineering [3,4,5]. Researchers have developed PLGA-based electrospinning nanofibers, PLGA-based nerve conduits, PLGA-based 3D-printed scaffolds, PLGA porous microspheres, and other scaffolds in various shapes for tissue regeneration and repair. However, PLGA is not hydrophilic enough to facilitate proper biointeractions, and there are no natural cell recognition sites on the surface of poly(lactide-co-glycolide) [6]. This limits the use of PLGA scaffolds in tissue repair and regeneration. As a result, functional coatings have been applied on PLGA scaffolds to increase cell adhesion and viability. These coating materials include polymers such as polyethylene glycol (PEG) [7], polydopamine (PDA), polypyrrole (Ppy) [8], poly-lysine [9], polyethyleneimine (PEI) [10], and ECM components such as HA [11], laminin [12], FN [13], and collagen [14]. Studies have shown that modifying PLGA-based scaffolds with these materials could improve the hydrophilicity and cell adhesion on PLGA. More importantly, differently modified materials and agents can confer PLGA scaffolds with additional favorable characteristics, which may further enhance their ability to promote tissue repair and regeneration.

There are a number of reviews about the modification of PLGA-based cell scaffolds. PLGA scaffolds were used widely as bone regeneration materials [15,16,17,18,19,20,21], through which the electrospun fibers for cartilage tissue regeneration [22,23] and the 3D-printed PLGA-based scaffold could prepare scaffolds with rich structure and function [24,25]. Moreover, PLGA combined some different materials could overcome the polymer’s limitations and extend its application in cell delivery and regenerative medicine [16,17,26,27]. In addition, modified PLGA-based scaffolds to deliver stem cells for nervous system repair [28,29,30,31] have been reviewed. There have been different scaffolds which are based on the mimicry of the extracellular matrix fabrication technique [32]. In addition, Pribadi et al. reviewed the correlation between nanotopography and dentin-pulp complex regeneration [33]. Lees et al.’ review described how 3D PLGA scaffolds seeded with stem cells and/or pancreatic progenitors may provide a benefit to achieving the normalization of blood glucose levels and summarized the problem of immune rejection of applying human stem-cell-derived islet progenitors in a clinical setting [34].

This review aims to summarize and discuss the advantages and disadvantages of the current coating materials used in PLGA scaffolds and intends to provide guidance in terms of selecting PLGA-based surface coating materials. The materials currently used to modify the surface of PLGA bio-scaffolds can be divided into organic and inorganic materials, and organic materials can be divided into synthetic polymeric materials, glycosaminoglycans, and bioprotein-based materials. The common features of the materials used for PLGA modification are as follows: they use their own structural characteristics to either improve the affinity and adhesion of cells to the scaffold, or regulate the behavior of cell proliferation, differentiation, and migration; they to participate in the process of cellular signaling; and PLGA mainly provides structural support and shapes the scaffold.

## 2. PLGA Scaffold Surface-Modified Methods

There are physical and chemical methods for PLGA scaffold surface modification. The physical methods include physical mixing, physical adsorption, electrostatic adsorption, electroplating coating, ultrasonic assisted coating, plasma-treated coating, and so on. The chemical methods include poly(ethylene-malonic acid) (PEMA) as a stabilizer, the generation of free radicals within the polymer scaffold structure, and chemical conjugation with proteins. This section will introduce some of the methods.

### 2.1. Physical Modification Methods

**Figure 1:**
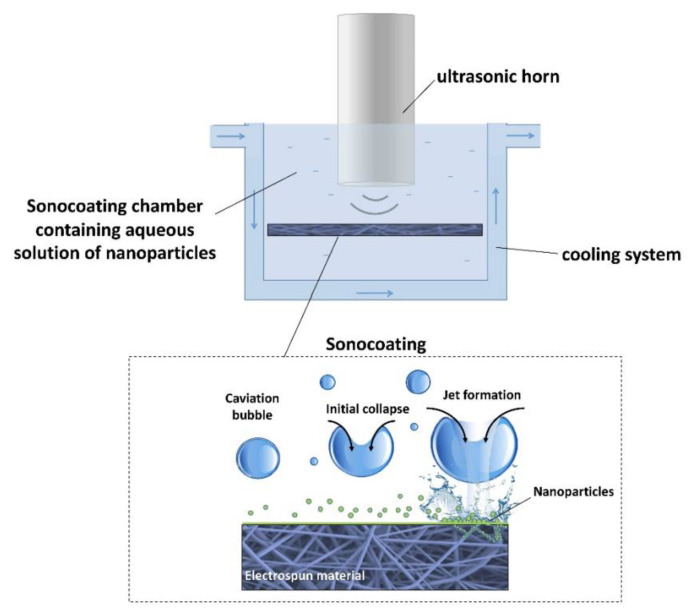
Schematic diagram of the ultrasonic coating setup and ultrasonic cavitation near the film surface during the process. Reprinted with permission from Ref. [39]. Copyright 2023, copyright Springer Nature, London, UK.

The principle of ultrasonic coating concerns the idea that system dynamics change significantly when acoustic cavitation occurs on a solid surface. As the environment is both uniform and pure liquid, the bubbles remain round as they collapse. Bubble collapses that occur near the solid surface in the solid–liquid system are very asymmetric, resulting in high-speed liquid micro-jets. These microjets eject the nanoparticle nuclei formed within the precursor solution in the direction of the solid substrate. These microjets are fast enough to adhere nanoparticles to solid surfaces [35,36]; in addition to the microjets, shockwaves begin to form. They have three times the energy of tiny objects and are thought to similarly promote and deposit microjets [37]. The ultrasound-assisted coating can deposit the prepared Ppy particles via PLGA spinning [38]. Additionally, in a sound-absorbing chamber, the researchers used sound waves to coat the surface of the PLGA film with nanohydroxyapatite (Figure 1) [39].

Physically mixing coating is a simple and effective way to deposit modified materials onto the surface of a PLGA, or to distribute it within a material after it has been modified. PLGA can also be uniformly mixed into solution. Studies have been performed where cell scaffolds are prepared via electrostatic spinning with the PEG/PLGA mixture [40]. This approach to coatings, where there are multiple layers, improves cell adhesion and viability by ensuring that the material surface remains hydrophilic during degradation. Dopamine self-assembles into a PDA coating on the PLGA scaffold surface. Self-assembling is an organic process (in the case of mercury, and possibly other liquid metals and alloys), and it is formed via the adsorption of molecular components from a solution or gas phase onto a solid surface, where it exists in a regular arrangement on a liquid surface; the adsorbent spontaneously (and sometimes epitaxially) organizes into a crystalline (or semicrystalline) structure [41].

**Figure 2:**
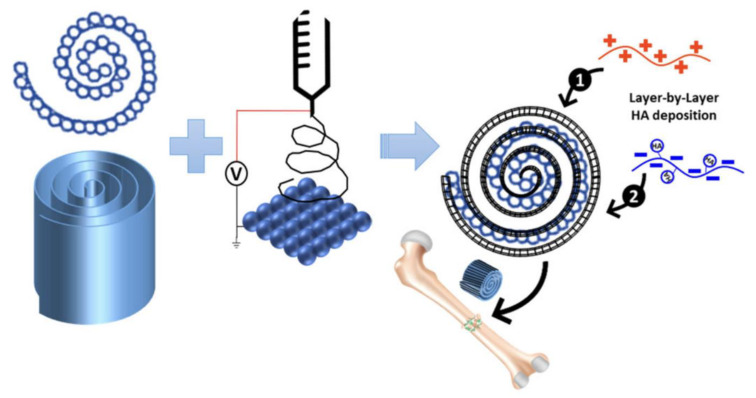
Preparing sintered PLGA microspheres and depositing electrospun nanofibers. The red is cationic chitosan and the blue is anionic HA. Reprinted with permission from Ref. [42]. Copyright 2023, copyright American Chemical Society, Washington, DC, USA.

Kumbar et al. [42] used a layer-by-layer (LbL) technique to distribute hydroxyapatite uniformly in PLGA scaffolds. LbL deposition is an effective and versatile technology that is based on electrostatic interactions between coordinative interactions and oppositely charged polymers (or hydrogen bonding) which produce multiple ultrathin layers from different materials. HA is deposited on a helical PLGA scaffold using an LbL polyelectrolyte process: first, it exhibits cationic chitosan (red), then anionic HA (blue), as shown in Figure 2. This process is repeated to obtain the desired number of HA bilayers. Zhao et al. [43] used LbL technology to produce type I collagen (Col I) and oxidized chondroitin sulfate (oCS) on the surface of PLGA in order to support inorganic mineral adhesion in a minimal physiologically based pharmacokinetic model that predicts anti-PEG IgG-mediated clearance of PEGylated drugs in human and mouse.

Similarly, PLGA scaffold surfaces have been treated with nitrogen plasma to ensure that they are negatively charged; indeed, they are immersed in a positively charged solution, then, they are immersed several times in a negatively charged solution comprising magnetic iron oxide in order to make the coating firm [44]. Both this method and the method which requires assembling multiple layers use electrostatic interactions between charges, and these are considered to be effective and simple methods that are suitable for coating any material with a charged surface.

### 2.2. Chemical Modification Methods

PLGA is synthesized using a ring-opening polymerization reaction, when PEG is added to the reactants and the synthesis conditions are adjusted, then PLGA-PEG or PLGA-PEG-PLGA can be obtained [45,46]. Covalent cross-linking is often used during protein coating in order to coat the PLGA scaffold. Researchers have shown that the pore size of gelatin [47] and collagen/HA [48] scaffolds decrease as the temperature also decreases; this is likely to be because of the effect of heat transfer rates on ice crystal nucleation and growth. Covalent immobilization, on the other hand, is a technique used for modifying the structure and texture of materials to improve the performance of porous scaffolds [49,50,51,52,53]. The surface of PLGA can be hydrolyzed with a NaOH solution, then, the carboxyl groups on the surface can be activated with 1-(3-dimethylaminopropyl)-3-ethylcarbodimide hydrochloride (EDC) and N-hydroxysuccinimide (NHS) solutions, and covalent crosslinker [53], laminin [49], and collagen [50]. Shulamit et al. used covalent immobilization to covalently bind tropoelastin to samples. Scaffolds were first treated by plasma immersion ion implantation (PIII), which generates free radicals within the polymer structure that propagate to form a highly reactive surface for irreversible biomolecule binding [54]. Moreover, hydroxyapatite can coat on the PLGA scaffold via the sputter deposition technique [55] or the ultrasound-assisted coating method after the PLGA surface has been treated with plasma.

## 3. PLGA Scaffold Surface-Modified Materials

### 3.1. Polymer Materials

#### 3.1.1. PEG

As a non-cytotoxic amphiphilic polymer, PEG can endow PLGA with good hydrophilic properties and it provides suitable conditions for cells to grow [56]. PLGA-PEG composite hydrogels or electrospun fibers have been used for post-myocardial infarction tissue regeneration [7], neural engineering [40,57,58], and skeletal muscle regeneration [59]. Liu et al. directly reprogrammed mouse embryonic fibroblasts (MEFs) into neural stem cells (iNSCs), and they demonstrated that the PLGA-PEG nanofiber scaffold was superior to the PLGA scaffold for iNSC adhesion and proliferation [58]. Furthermore, after the transplantation of iNSCs-seeded PLGA-PEG scaffolds into a rat spinal cord transection injury model, cells’ differentiation into glial cells and neurons, in addition to better survival rates, was observed. The PLGA-PEG surface increased cell viability over time compared with PLGA, and both functional connectivity and synaptogenesis were induced in the cells plated on the PLGA-PEG surface. This coincided with the increase in synaptophysin and synapsin-1, which did not occur in the PLGA and control groups.

PEG was able to form a hydrated film via hydrogen bonding with water molecules; however, this is not conducive to adsorbing proteins [60]. The addition of other materials to PLGA-PEG scaffolds increased cell adhesion; for example, adding gelatin increased the adhesion of dental pulp stem cells [61]. In addition, studies have shown that the body’s ability to recognize and bind with anti-PEG antibodies may be a challenge for PEG-functionalized PLGA stents in vivo in the long term [62]. Various elements such as cells [63], cytokines [64], therapeutic drugs [65], or inorganic nanomaterials [7] can be wrapped into the hydrogel in the sol-to-gel state to regulate the physiological functions of cells and enhance cell–tissue communication and integration while these elements are delivered to cells.

#### 3.1.2. Polydopamine (PDA)

**Figure 3:**
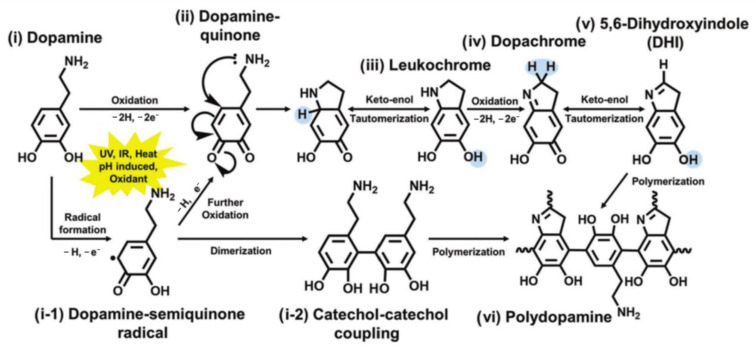
Formation of PDA: A copolymer of 5, 6-dihydroxy indole (DHI) and dopamine. Reprinted with permission from Ref. [83]. Copyright 2023, copyright John Wiley and Sons, Hoboken, NJ, USA.

Polydopamine (PDA), a biomimetic polymer, holds promise in the realm of chemical patterning as it augments adhesion and direct cell growth. Remarkably, PDA exhibits a strong affinity for virtually all solid surfaces, irrespective of their surface chemistry, and thus, it is able to form a thin, biocompatible, and hydrophilic layer. Notably, studies have shown that PDA can facilitate the adhesion and proliferation of various mammalian cells, rendering it a potential candidate for utilization in the field of tissue engineering [66]. PDA could promote the hydrophilicity of PLGA scaffolds and provide cell recognition sites for cell adhesion. PDA coating could facilitate cell adhesion and proliferation and affect cell fate. PDA was synthesized via the self-polymerization of dopamine in an alkaline solution. Dopamine quinone was formed by autoxidizing dopamine, which was then cyclized to form the PDA precursor dihydroxyindole (DHI) [67]. The process of PDA formation is shown in Figure 3. PDA contains abundant functional groups which make it hydrophilic, such as catechols, quinones, imines, amine functional groups, and *π* conjugated structures [68]. Additionally, PDA can be deposited very easily on almost all types of organic and inorganic substrates, including super-hydrophobic substrates [69]. The synergistic effect of the amine and catechol may be responsible for the strong adhesion of PDA to various materials [70,71]. PDA has been widely used in antibacterial materials [72], nerve tissue repair [73,74], skin repair, bone defect repair [3,75,76,77,78,79,80,81], periodontal tissue regeneration [82], and other fields due to its excellent properties.

**Figure 4:**
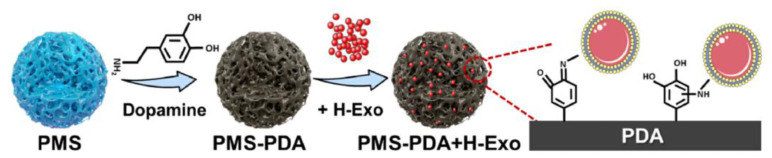
Porous PLGA microspheres coated with PDA so that they may act as a carrier for exosomes. Reprinted with permission from Ref. [3]. Copyright 2023, copyright Elsevier, Amsterdam, The Netherlands.

PLGA scaffolds with a PDA coating have good lipid and protein adsorptive properties. Gao et al. [3] added 2 mg/mL of dopamine to a Tris HCl solution with a pH of 8.5, and PDA was coated onto PLGA porous microspheres at a low rotation speed in order to prepare PLGA-PDA porous microspheres. These PMS-PDA microspheres were able to effectively adsorb exosomes, and they showed a high exosome retention rate at the treatment site. Moreover, they sustained the release of exosomes for 21 days and effectively induced the vascularized bone regeneration of cranial defects in rats (Figure 4). Through the adsorption of exosomes, cells, and other biological products, PDA coated on a PLGA scaffold has the potential to enhance cell adhesion.

The PDA coating can also improve protein stability. Yang et al. [84] immobilized platelet-derived growth factors on the PLGA fibers which had a PDA coating in order to improve the healing of skin defects on the back, promote angiogenesis, and reduce inflammatory cytokines levels in rats. PLGA-PDA scaffolds were also applied to cell carriers. Zhang et al. [85] prepared a PLGA scaffold via the electrospinning method and modified it with a polydopamine coating. The PDA-PLGA scaffold promoted angiogenesis and it exhibited a good biological safety performance. Simultaneously, the PDA-PLGA scaffolds did not affect the secretory function of the islet cells. PDA-PLGA scaffolds with RINm5f cells were implanted into the skeletal muscle of type I diabetic rats. After one week, the blood glucose levels in the treatment group were significantly lower than those in the model group. This suggests that the skeletal muscle graft site might be a potential new choice for future islet cell transplantation. However, under oxidative conditions, the phenolic groups in the PDA coating were converted to quinone groups, which subsequently reacted with molecules containing a mercaptan or amino group [86]. As a result, human induced pluripotent stem cells could not adhere and proliferate on the PDA surface [87], and neuronal cells did not adhere well with PDA [88], possibly because the PDA membrane bonds strongly to sulfhydryl, amine, and imidazole groups [86]. However, the properties of various structures and functional groups on the PDA surface could be used to deliver proteins in order to improve PDA coating when used for tissue regeneration. Alternatively, the surface of the PDA can be modified to enhance its original function or to give it a new function. Some modification methods and their potential applications in cell adhesion culturing and tissue engineering are listed in the following table (Table 1).

**Table 1:** PDA Surface modification methods and applications.

PDA Surface Modified Type	Method of Modification	Function	Application	Time	Ref.
PDA co-precipitated with PEI	Amine groups in PEI, such as primary, secondary, and tertiary amines, can react with PDA	Support neuronal adhesion PC12	Preparation of cell models, biosensors, diagnostics, and tissue engineering products	2012	[89]
Co-precipitation of PDA with galactose-conjugated PEI	The amine group in PEI can react with PDA	Support HepG2/C3A hepatocyte attachment	Preparation of cell models, biosensors, diagnostics, and tissue engineering products	2012	[89]
Grafting of polymers such as polymethyl methacrylate, polymethyl acrylate, poly(4-pyridyl acetate), and poly(tert-butyl acrylate) onto PDA nanosheets by self-consistent photo-grafting and photopolymerization	UV can promote hydrogen in the amino and hydroxyl functional groups in PDA nanosheets to generate free radicals and subsequently initiate polymerization reactions	PDA nanosheet with a hydrophobic layer	Preparation of cell models, biosensors, diagnostics, and tissue engineering products		[90]
Chitosan derivative carboxymethyl chitosan as a linker for vertical and controlled attachment of VN peptides to the synthetic PDA surface	The PDA-modified surface was bound to the amino- and carboxyl-containing biomolecule carboxymethyl chitosan via Michael addition and Schiff base reactions between the amino and catechol/quinone groups, followed by reaction with the standard NHS/EDC chemistry for VN peptides (Figure 5).	Promotes reprogramming of human induced pluripotent stem cells into fibroblasts, and promotes induced differentiation into cardiac myocytes and neuronal growth.	Cell culture vector, tissue engineering	2016	[87,91]
Cross-linked bone morphogenetic proteins	Cross-linked bone morphogenetic proteins	Promote the adhesion and proliferation of MC3T3-E1 cells, promote the activity of phosphatase, the mRNA expression of osteogenic genes, and the deposition of calcium.	Promote bone regeneration	2017, 2018, 2019	[79,81,92]

**Figure 5:**
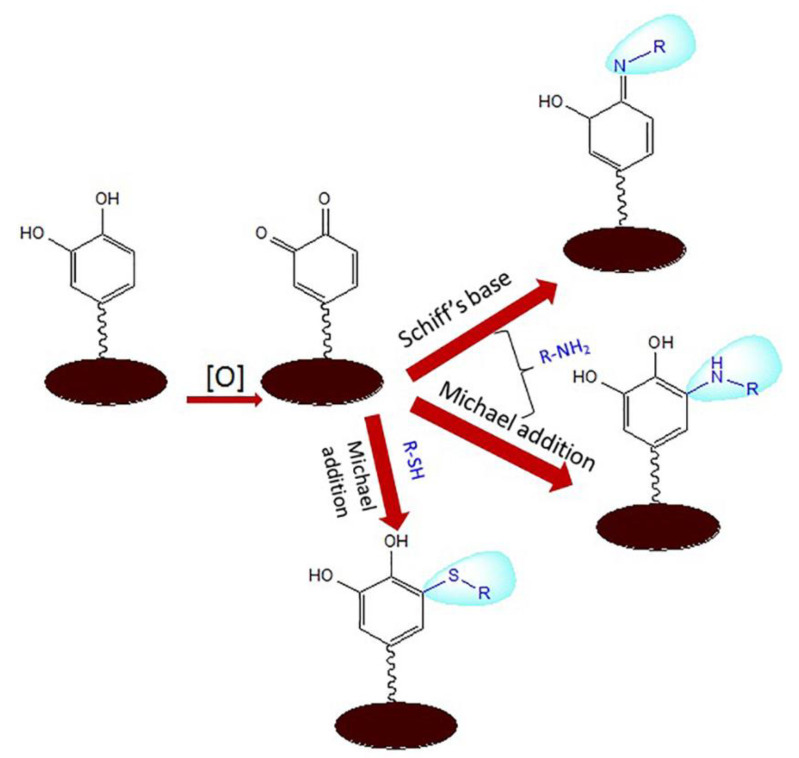
Michael addition and Schiff base reactions of reactive aminating and sulfurylating ligands. Reprinted with permission from Ref. [91]. Copyright 2023, copyright Frontiers, Lausanne, Switzerland.

#### 3.1.3. Polylysine

**Figure 6:**
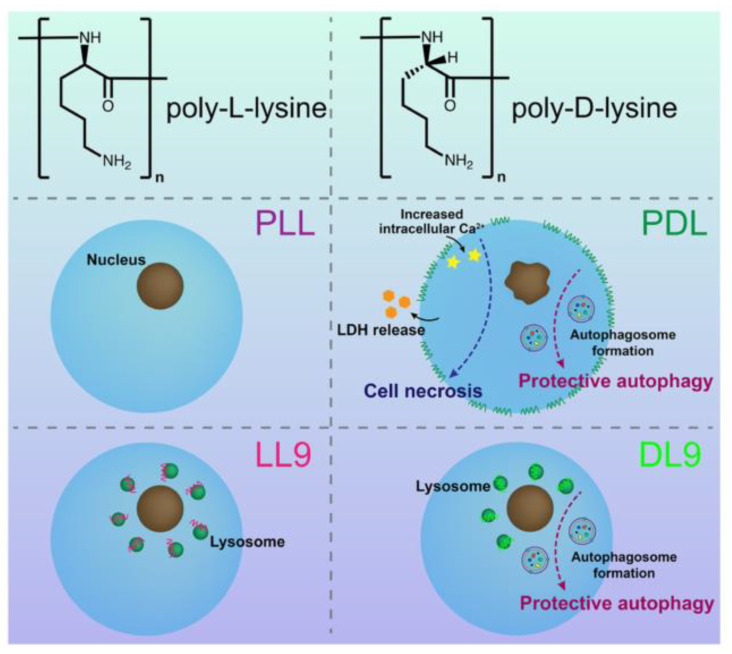
Schematic representation of different cell responses when induced by PLL, PDL, LL9, and DL9. Reprinted with permission from Ref. [9]. Copyright 2023, copyright America Chemical Society, Washington, DC, USA.

Polyamino acids have long been used for tissue repair [93]. Polylysine could change the electric potential of the PLGA scaffolds surface to positive potential, improve hydrophobicity of PLGA scaffolds, promote cell adhesion, and adjust cell behaviors. Polylysine is a peptide formed by lysine polymerization. The amine-containing side chain of polylysine can carry a strong positive charge in an aqueous solution and it can enhance cell adhesion via electrostatic adsorption and hydrogen bonding [94]. Due to the chirality of polylysine, it can be divided into poly-D-lysine (PDL)and poly-L-lysine (PLL). PLL is a polypeptide that was first discovered by Japanese scientists, and it was found in Streptomyces albicans in 1977 [95]. PDL is the right-hand body of synthetic PLL. PLL and PDL can induce different cell responses (Figure 6).

When used as a scaffold coating for tissue engineering, PLL mainly improves neuronal adhesion and promotes peripheral nerve repair. Davood Zolfaghari et al. [96] determined that the hydrophobicity of PLGA coated with PLL significantly increased the contact angle studies, and the level of cell proliferation was increased when nanofibers coated with PLL were used.

PDL together with collagen may be used to modify PLGA microspheres, and as a vector for the delivery of human-induced pluripotent stem cells (hiPSC-CMs) into the heart [97]. Results have shown that this vector could increase cell growth and the cell survival rate by 1.70-fold to 1.99-fold after one day and four days, respectively. In vivo, the results showed that these cells not only maintained their cardiac phenotype but they also showed signs of in vivo electrical coupling and maturation, which importantly improved cardiac function. These examples demonstrate the potential of polylysine-coated scaffolds as cell carriers for tissue regeneration. It must also be noted that the surface properties of PLGA microspheres can impact the effect of the coating. When propylene glycol is used instead of polyvinyl alcohol as the required surfactant during the preparation of PLGA microspheres, the coating distribution of the poly(D-lysine/fibulin) mixture and the subsequent attachment of MSCs are affected [98].

Furthermore, a high-molecular-weight (Mw) poly-L-lysine (27.4 kDa, 36.6 kDa) may affect the metabolic activity of cells and it has a specific cytotoxicity [99], which is related to the inhibition of mitochondrial function [100]. In addition, Wang et al. [9] found that PDL could be anchored to the plasma membrane and interact with membrane lipids, which led to the rapid morphological change and death of A549 cells (a human lung cancer cell line), as well as HPAEpiCs (a human pulmonary alveolar epithelial cell line). However, PLL was not anchored to the plasma membranes of these cells and thus it exhibited good cytocompatibility. PDL was able to trigger protective autophagy to protect cells to some degree, and the PDL-caused cell death occurred via intense necrosis (due to increased intracellular Ca^2+^ content and plasma membrane disruption). Additionally, studies note that short-chain PDLs, with a repeat unit number of 9 (termed DL9), could localize to lysosomes and induce autophagy at high concentrations, but they cannot induce drastic cell death, proving that the repeat unit number of polylysine could influence its cellular action. The effect of high Mw PLL on cell viability differed from the results of the abovementioned studies, which may be due to the use of different cell lines. Further studies should clarify the specific reasons for this phenomenon.

#### 3.1.4. Polypyrrole (Ppy)

**Figure 7:**
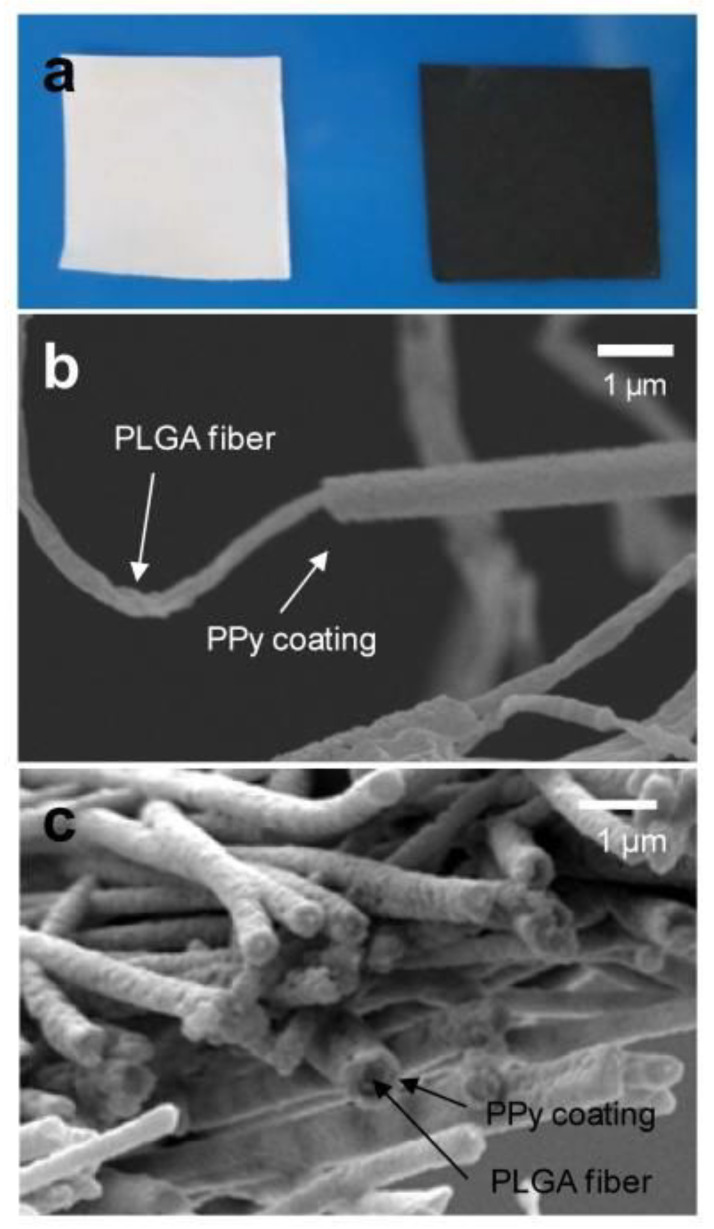
Ppy-coated PLGA grids. (**a**) Photos of uncoated PLGA grids (white, left) and Ppy-PLGA grids (black, right); (**b**) SEM micrograph of a single strand of Ppy-PLGA fibers. (**c**) SEM image of section of the PPy-PLGA meshes. Reprinted with permission from Ref. [38]. Copyright 2023, copyright Elsevier, Amsterdam, The Netherlands.

**Figure 8:**
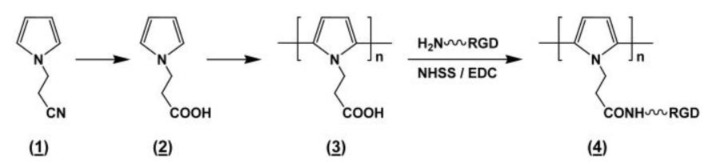
Synthesis scheme of a cyanide-functional pyrrole (**1**), a carboxy-functional pyrrole (**2**), a carboxy-functional Poly(1-(2-carboxyethyl)pyrrole (PpyCOOH) (**3**), chemically coupled with RGD peptide, RGD grafting PpyCOOH (**4**). Reprinted with permission from Ref. [116]. Copyright 2023, copyright American Chemical Society, Washington, DC, USA.

Ppy is a conductive biomaterial that has been used to repair nerves [101] and myocardium [102]. Ppy coating could facilitate the cell adhesion, proliferation, and differentiation of some cells (the structure of Ppy is shown in Figure 8). Studies have shown that passing electrical stimulation through Ppy could enhance skeletal muscle differentiation, cell proliferation [103], bone regeneration [104,105], myocardial repair [8,106,107], and nerve repair [38,108,109,110]. Lee et al. [38] produced electroconductive tissues by growing Ppy on randomly arranged electrospun PLGA nanofibers, and neuronal growth and axon formation increased as a result of electrical stimulation (Figure 7). Moreover, coating the surface of PLGA fibers with Ppy to create an electromechanically active fiber scaffold could achieve the mechanical and electrical stimulation of iPS cells. This dual stimulation facilitated the differentiation of iPS cells into cardiomyocytes without affecting cell viability and morphology [111].

**Figure 9:**
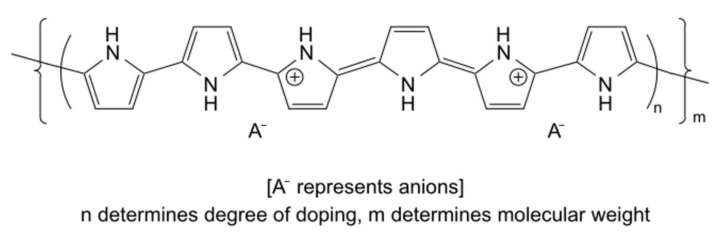
Structure of Ppy with the dopant (anion) A^−^. Reprinted/adapted with permission from Ref. [103]. Copyright 2023, copyright Elsevier, Amsterdam, The Netherlands.

Cell adhesion can be further enhanced by adding carboxyl-functionalized Ppy; for example, poly(1-(2-carboxy-ethyl)pyrrole) (PpyCOOH) post-graft RGD has been synthesized (Figure 8) [112], and dopant molecules may be added to modified polymers (Figure 9). The high binding affinity of Ca^2+^ to the catechol moiety of doped dopamine could accelerate the deposition of hydroxyapatite in a simulated body fluid [113]. A novel Cu-doped Ppy with trienzyme-like activities [114], and a highly conductive, soft, biocompatible conducting polymer hydrogel (CPH) based on a plant-derived tannic acid (TA), as well as polyphenol, are able to create cross-linking Ppy chains that can conduct doping and stimulate tissue repair for a spinal cord injury (SCI) [115].

The disadvantages of using Ppy as a conductive scaffold coating have not been identified. However, the Ppy process itself is poor in terms of mechanical properties, and the products exhibit poor adhesion, as well as insolubility in water and most organic solvents, which makes processing complex [117]. Moreover, they also have a poor electrical conductivity due to uneven distribution on the scaffold surface [118]. To address this, Weng et al. [119] prepared inkjet-printed Ppy films with good conductivity and uniformity using a printable Ppy nanodispersion tool; these films were stable, and the researchers demonstrated the cytocompatibility of these platforms using PC12 cells. Zhang et al. [117] found that DA functionalization enhances the stability and dispersibility of Ppy in water, and its film adhesion to the surface of the substrate improved significantly. However, they also found that high concentrations of Ppy (>9.7 µg/mL) are detrimental to the proliferation and viability of cells [118]. Conversely, He et al. [118] obtained spherical methyl acrylic anhydride-gelatin (GelMA)-Ppy nanoparticles when GelMA methacrylate groups formed a self-crosslinked network via the oxidative polymerization of Ppy, and the GelMA-Ppy nanoparticles showed excellent biocompatibility at a high concentration of 50 mg/mL. After modifying or combining with other materials, the cytotoxicity of Ppy is greatly reduced.

#### 3.1.5. PEI

PEI coating can enhance cell adhesion on PLGA scaffolds. PEI is a cationic polymer commonly used for gene delivery [10]. It has also been modified on PLGA porous microsphere cell carriers to deliver MSCs to the site of infarction for the treatment of myocardial infarction [6]. However, relevant studies have pointed out that attention should be paid to the cytotoxicity of PEI due to its linear structure and high electron density which cause cellular necrosis [99]. The cytotoxicity of polycations is directly related to Mw and pKa. Materials with a higher Mw and more cations are more toxic, and even low Mw polycations might disrupt cell membranes over time, indicating that there is no such thing as “non-toxic” polycations [120]. However, it has been reported that extracellular vesicles derived from MSCs, which are released from PEI surface-modified PLGA materials, could reduce the inflammation of endothelial cells and increase angiogenesis [121].

### 3.2. Mimics ECM Materials

The ECM provides a stable 3D environment wherein cells can attach, grow, proliferate, migrate, and differentiate. Researchers have combined ECM components that lack mechanical strength with hydrophobic PLGA scaffolds that comprise insufficient cell adhesion conditions in order to mimic the environment in which cells grow in the body. Components that are currently being applied to PLGA-based cell scaffold coatings include the following: HA, gelatin, collagen, laminin, and FN. They have similar functions of promoting cell adhesion, proliferation, and activity and regulating cell fate.

#### 3.2.1. HA

HA, a nonsulfated glycosaminoglycan, is an important natural ECM, and it is composed of repeating units of β (1,3)-D-glucuronic acid and β (1,4)-N-acetyl-D-glucosamine. The natural polymer is a component of the ECM of all connective tissues, and it comprises functions related to cell signaling, wound repair (in which cell membrane receptors are directly involved in cell-HA interactions), and stromal tissue functions [122]. HA is hydrophilic and can be rapidly degraded in vivo via hyaluronidase [123] or hydrolysis [124]. Researchers have found that L929 fibroblasts on 85:15 ester-linked PLGA scaffolds exhibit the highest level of cell viability, compared with 50:50 acid-linked and ester-linked PLGA scaffolds [125]. The HA coating can also enhance the adhesion and proliferation of MSCs; therefore, it can be used to repair bone [126]. Wojak-Cwik et al. [127] demonstrated that MSCs which were cultured on porous PLGA scaffolds, and coated with type I collagen and high-sulfate hyaluronic acid (sHya), could enhance proliferation and alkaline phosphatase activity. They also demonstrated that mineralization of MSCs, and the expression of Runt-related transcription factors, such as osteopontin, and bone salivary protein II were enhanced [126], thus indicating good osteogenic differentiation.

#### 3.2.2. Gelatin

As a derivative of collagen, gelatin is non-cytotoxic and it possesses a good electrical conductivity. Gelatin can regulate cell adhesion, initiate some biological responses, and promote faster cell healing via the integrin α2β1 signaling pathway due to the rich RGD-like sequence in the polymer chain of gelatin fibers [128,129]. It has been used as a PLGA coating in nerve repair [96,130]. Gelatin has also been widely used as a PLGA scaffold coating in bone tissue engineering [53,96] and skin repair [131]. Dou et al. [53] demonstrated that the gelatin network in scaffolds acted as an ECM in the early stage of transplantation, but with increased cell adhesion. Pranke et al. [127] configured a powerful 3D scaffold using conduits coated with gelatin, which ensured very good stem cell adhesion, viability, and proliferation. The gelatin treatment promoted an increase in cytoskeletal activity compared with PLGA-only conduits, and the cell proliferation and viability of the PLGA/gelatin conduits increased, as compared with the PLGA-only conduits.

**Figure 10:**
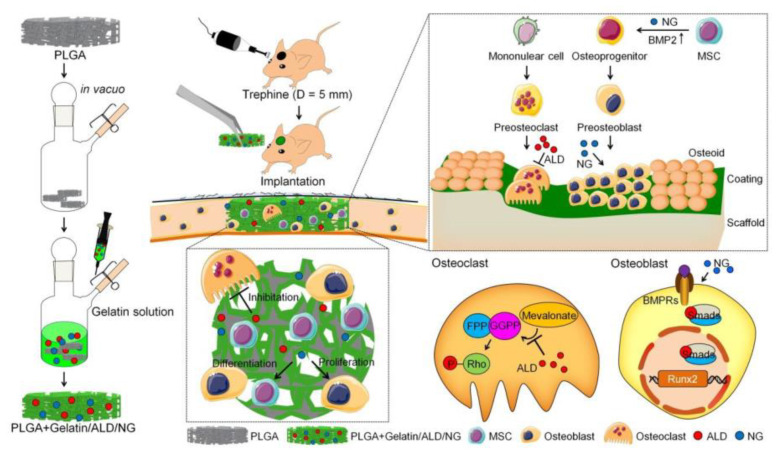
PLGA + gelatin/ALD/NG stent implantation to repair a cranial defect in rats. Reprinted/adapted with permission from Ref. [124]. Copyright 2023, copyright America Chemical Society, Washington, DC, USA.

Zhu et al. [128] demonstrated that the mechanical strength and hydrophilicity of PLGA scaffolds were improved via the addition of sodium alendronate (ALD) and naringin (NG) to the gelatin coating (Figure 10). Interestingly, ALD had an inhibitory effect on osteoclast activity, and NG had an osteogenic effect on MSCs; the two drugs exhibited a synergistic effect on the repair of cranial defects in rats (Figure 10). The PLGA stent with gelatin, ALD, and NG (PLGA + gelatin/ALD/NG) almost completely repaired the physiologically intact rat skull defect at 16 weeks, as per the results of histopathologic staining and microscopic computed tomography.

#### 3.2.3. Collagen

**Figure 11:**
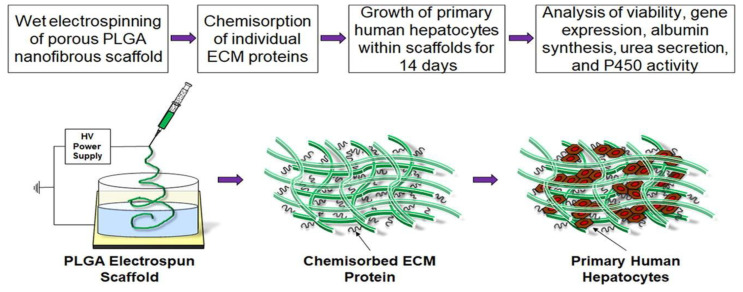
Preparing the PLGA-ECM scaffold. Reprinted/adapted with permission from Ref. [131]. Copyright 2023, copyright Elsevier, Amsterdam, The Netherlands.

Collagen is the most abundant protein in the mammalian body, and it comprises approximately 30% percent of the body’s total protein content. Collagens play a structural function in that they support the mechanical properties, organization, and shape of various tissues. They regulate cell migration, proliferation, and differentiation via interaction with cells through several receptor families [52,132]. Type I collagen is the most plentiful and well-studied type of collagen. It comprises more than 90% of the organic mass of bone, and it is the most important collagen in skin, tendons, ligaments, corneas, and many interstitial connective tissues, except for some tissues in the brain and, hyaline cartilage, as well as vitreous tissue [133]. Yong et al. [134] found that a PLGA scaffold with a cell-derived extracellular matrix (CDM) was able to provide blood-derived mesenchymal stem cells (UCB-MSCs) with a better microenvironment for osteogenesis in vitro. Furthermore, type I collagen was chemically linked to electrospun PLGA, derived from amine coupling; this type of collagen could thus develop an in vitro culturing system that contains the minimum set of essential ECM components found in the liver micro-environment [135]. Jessica et al. [135] demonstrated that the incorporation of type I collagen into PLGA scaffolds (PLGA-Chigh: 100 µg/mL) resulted in a 10-fold higher albumin secretion, 4-fold higher urea synthesis, and increased transcription of hepatocyte-specific CYP450 genes (CYP3A4, 3.5-fold increase, and CYP2C9, 3-fold increase) in primary human hepatocytes as compared with the same cells grown in unmodified PLGA scaffolds for two weeks (Figure 11).

#### 3.2.4. Laminin

**Figure 12:**
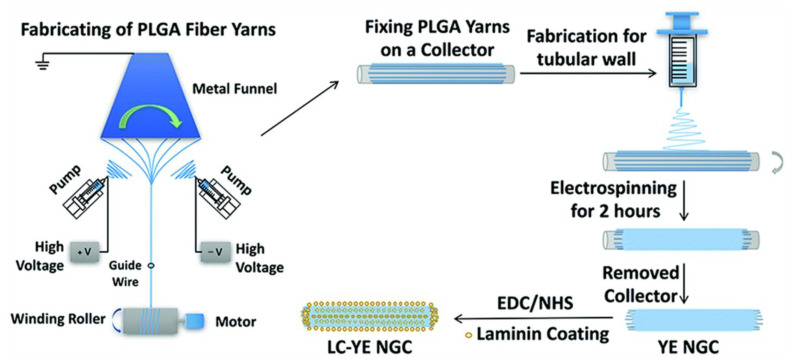
Adhesive-coated PLGA (LC-YE- PLGA) NGC (YE NGC: Yarn-wrapped neural guiding catheter; LC-YE NGC: Laminin-coated yarn-coated neural guiding catheter). Reprinted/adapted with permission from Ref. [49]. Copyright 2023, copyright Royal Society of Chemistry, London, UK.

Laminin, an ECM glycoprotein, interacts with receptors anchored on the plasma membrane that is adjacent to the basement membrane; this is one of its most significant functions. Laminin interacts with various cell receptors, including integrins, poly ligand proteoglycans, α-dystrophins, and the Lutheran/basal cell adhesion molecule (Lu/B-CAM). It adheres cells to the ECM by binding to integrins on the cell membrane. During the process, laminin regulates various cellular activities and signal transduction pathways [136] which can benefit nerve regeneration (Figure 12) [12,49,137,138].

Further research has shown that laminin with a PDA coating enhances the effectiveness of synaptic extension promotion [137]. In vivo experiments have demonstrated that a laminin-coated PLGA nerve catheter can not only promote the generation of sciatic nerve fibers [12], but it may also incorporate growth factors and adipogenic stem cells that promote the regeneration and functional recovery of the sciatic nerve [138]. In addition, Hoareau et al. [139] used a laminin-coated PLGA microcarrier to deliver stromal vascular fraction (SVF) cells to the ischemic limb of a diabetic mouse model. The results showed that the delivering of cells via a microcarrier was more beneficial in terms of improving blood flow, reducing necrosis, and restoring limb sensation than injecting cells alone.

#### 3.2.5. FN

FN plays a vital role in cell adhesion, growth, migration, and differentiation as it mediates various cell ECM interactions. FN is involved in a wide range of functional activities, and it binds to the cell surface through integrins. It also binds to some biologically important molecules, including collagen/gelatin, heparin, and fibrin, and these interactions are mediated by a number of different structural and functional domains [140]. Mobasseri et al. [2] demonstrated that MSCs cultured on an FN-coated substrate adhere well in a pattern after 2 h of incubation. Li et al. [141] found that neural stem cells (NSCs) spheres and fibroblasts grew in an aligned pattern along the direction of the fibrin fibers, and the NSC spheres were able to grow and differentiate into neurons on the fibrin and PDL-coated aligned collagen-PLGA composite scaffold. Kaufman et al. [142] found that fibrin-coated PLGA nanofibers induced cell migration toward the fibers, and they supported cell growth within the scaffolds. They also influenced the spatial rearrangement of fibroblasts by promoting packed cell clusters or intermittent cell spreading. Furthermore, these cell arrangements were similar to the structural characteristics of dense and soft connective tissues, respectively. Long-term transplantation experiments in animals showed good biocompatibility [13], thus suggesting that FN-coated PLGA scaffolds have the potential to promote the healing of wounds.

#### 3.2.6. Bionic Cell Membrane

Cell membranes that are coated in materials such as red blood cells, platelets, white blood cells, cancer cells, bacteria, and so on, can effectively improve PLGA surface biocompatibility [143]. Cell membranes comprise part of the organism, and thus they do not trigger immune responses. Short-term inflammatory responses, such as neutrophil infiltration and proinflammatory cytokine upregulation, were eliminated when PLGA-based materials coated with cell membranes were implanted in vivo [144]. The cell-membrane-coated PLGA scaffold is similar to PLGA surfaces that are modified with proteins of the ECM [145,146]. The cell-membrane-modified PLGA surface is designed to increase cell adhesion and regulate the activity and differentiation of adherent cells through cell membrane surface proteins. The differentiation of pancreatic stem cells into insulin-secreting cells can be induced by culturing pancreatic stem cells on fibroblast-modified PLGA membranes [145]. The results showed that fibroblasts improved PLGA membrane cytocompatibility and histocompatibility, and they also promoted pancreatic stem cell proliferation and differentiation. After induction, the number of Notch receptors and their ligands expressed on the cell membrane of pancreatic stem cells was higher than that of non-induced pancreatic stem cells or fibroblasts, as shown by the real-time fluorescence quantitative PCR results. Semiconductor, quantum dot-coupled, anti-complex probe experiments showed that induced pancreatic stem cells exhibited a higher expression of Notch 2 and Delta-like 1 than non-induced pancreatic stem cells, and this potentially occurred due to the Notch signaling interaction between fibroblasts and pancreatic stem cells. The proliferation of pancreatic stem cells and their differentiation into insulin-secreting cells is promoted by regulating the neurogenin-3 (Ngn3) and mitogen 1 gene expression via Hairy/Enhancer of split-1 [142].

**Figure 13:**
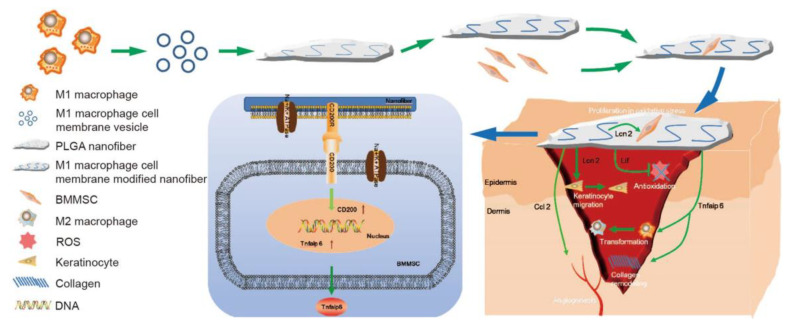
Diabetic wound repair via the LPS/IFN-γ activation of mouse RAW264.7 cell membranes. Reprinted/adapted with permission from Ref. [142]. Copyright 2023, copyright Springer Nature, London, UK.

Gao et al. [146] modified PLGA nanofibers using LPS/IFN-γ-activated macrophage cell membranes (Figure 13). The modified fibers promoted bone marrow (MSCs) proliferation and keratinocyte migration under oxidative stress in vitro. Furthermore, bone marrow mesenchymal stem cell-loaded fibers accelerated wound healing, which was accompanied by rapid epithelial regeneration, antioxidant stress, collagen remodeling, and angiogenesis; this occurred during experimental diabetic wound healing. Transcriptome analysis showed that when bone marrow MSCs were co-cultured with modified fibers, the expression of wound-healing-related genes was upregulated.

#### 3.2.7. Silk Fibroin

A natural protein can improve the cell adhesion and hydrophilicity of PLGA scaffold and ECM proteins. Silk fibroin is a natural protein that is synthesized and secreted by silkworms and spiders. In some studies, the amino acid sequence of silk fibroin protein has been found to enhance cell adhesion and activity. Moreover, it is an excellent natural polymer that can be used in bioengineering because of its good biocompatibility and biodegradability [147]. The composite scaffold, prepared by the silk fibroin, PLGA, and ECM mixture, can be used in tissue engineering. It was found that the scaffolds containing 50% of PLGA, 25% of fibroin, and 25% of collagen exhibited high porosity and good hydrophilicity, and as such cells could adhere, grow, and proliferate [148]. The hydrophobicity of the PLGA scaffolds was also improved by introducing fibroin, and the adhesion and vitality of the chondrocytes and fibroblasts cultured on PLGA scaffolds were significantly enhanced [149]. The results of the animal experiments and histopathologic evaluation showed that the residual wound area of the PLGA/SF (2:1) mixed membrane group was significantly smaller than that of the PLGA group and the control group, thus suggesting that this composite material could be used as a dressing for chronic wounds [150]. Moreover, the scaffold can cultivate human umbilical vein endothelial cells [147].

### 3.3. Inorganic Material

#### 3.3.1. Magnetic Material

Magnetic-material-coated PLGA scaffolds could induce cell proliferation, differentiation, and promote tissue repair. Biocompatible and self-degrading magnetic iron oxide nanoparticles can promote stem cell differentiation into bone cells in vitro [44,151] and bone regeneration in vivo [152,153,154]. Their application on the PLGA scaffold surface can regulate oral flora and promote bone regeneration [155]. The PLGA composite coating of antibacterial silver nanoparticles combined with superparamagnetic iron oxide prevents bacterial adhesion to the scaffold and promotes osteoblast proliferation [156]. Hao et al. [157] prepared a scaffold using oleic acid-modified iron oxide nanoparticles (IO-OA NPs) which generated PLGA-based homogeneous magnetic nanocomposites in order to regulate cell behavior and promote bone regeneration. In the presence of SMF in vitro, cell attachment and osteogenic differentiation were significantly enhanced by the IO-OA/PLGA composites, as indicated by increased alkaline phosphatase activity, enhanced mineralization nodule formation, and upregulated bone-related gene expression (phosphatase, OCN, and BMP2), in a dose- and time-dependent fashion. Furthermore, the synergistically enhanced osteogenic differentiation primarily occurred due to mechanical stimuli, as indicated by the upregulated expression levels of piezo-type mechanosensitive ion channel component 1 (Piezo1), a key receptor for sensing mechanical stimuli.

#### 3.3.2. Hydroxyapatite

Hydroxyapatite could promote cell adhesion and bone regeneration. Hydroxyapatite is a calcium phosphate mineral found in mammalian teeth, vertebrate bones, fish scales, and some beetle adult teeth. It is the primary mineral component of the skeletal system of vertebrates and has been widely used in tissue engineering because of its good biocompatibility and biodegradability [158]. In addition, the PLGA/hydroxyapatite composite can be used to improve collagen’s thermal and conformational stability, as it is a type of material that can be used in tissue engineering because of its non-toxicity to cells and degradable nature [159]. Studies have shown that hydroxyapatite-coated PLGA scaffolds can support human cord-blood-derived stem cells in terms of their ability to adhere, proliferate, and undergo osteogenic differentiation [160].

Furthermore, MSCs proliferation and osteogenic differentiation were enhanced on hydroxyapatite-coated PLGA scaffolds [161,162]. The study showed that nano-sized hydroxyapatite has a more robust adsorption capacity on cells, and the roughness of the hydroxyapatite surface has a particular effect on cell adhesion [163]. PLGA scaffolds with hydroxyapatite coatings have been widely used in bone repair [164,165,166]. Despina et al. [163] found that cell adhesion, detachment strength, and proliferation were surface roughness sensitive, and they became elevated as the roughness of the hydroxyapatite increased and the percentage of adherent cells decreased sigmoidally with applied shear stress. Chou et al. [167] found that cell responses differed when subtle changes occurred in the apatite microenvironment. Micro- and nano-hydroxyapatite scaffolds have the potential to promote the adhesion and activity of adipose stem cells, enhance the activity of alkaline ALP activity, increase the mRNA expression levels of osteogenic markers and angiogenesis factors, and promote osteogenesis and angiogenesis in vivo [168].

#### 3.3.3. Bioactive Glass

Bioactive glasses could promote cell adhesion and bone regeneration. Bioactive glasses have been successfully used for bone regeneration because of their excellent bioactivity and ability to bind to bone tissue. Their degradation rate in vivo matches that of bone formation, and through the combination of surface apatite crystallization and ion release, they stimulate osteocyte proliferation, thereby forming new bone [1,169]. Recent studies have shown that bioactive glass also promotes blood vessel formation and wound healing [170,171]. The most commonly cited commercial active glass, 45S5, comprises 45% SiO_2_, 24.5% Na_2_O, 6% P_2_O_5_, and 24.5% CaO. Bioactive glass-coated PLGA scaffolds have been used to increase the adhesion of bone marrow-derived MSCs and to help them to differentiate into bone cells [172].

#### 3.3.4. Graphene

Graphene could promote cell adhesion, proliferate, and differentiation. Graphene, which consists of a single layer of carbon atoms arranged in a 2D honeycomb lattice [173], is a very useful nanomaterial for biomedical applications due to its electrical conductivity, biocompatibility, excellent flexibility, high strength, stiffness, and thermal properties [174]. Recently, scientists have used graphene for stem-cell-based tissue engineering because of its physicochemical properties and biocompatibility [175]. Graphene has been shown to help to proliferate and differentiate between adult and pluripotent stem cells [176,177]. Graphene oxide (GO) is a chemically versatile nanomaterial with oxygen functional groups attached to the graphene substrate. Moreover, its surface is rich in oxygen-containing groups such as hydroxyl, epoxy, and carboxyl groups, thus making it hydrophilic, which promotes cell adhesion [178,179,180]. For the selective differentiation of neural stem cells (NSCs) into oligodendrocytes, some studies have demonstrated that GO, in combination with electrospun nanofibers, is an effective coating material [179].

## 4. Modified Material Problems and Solutions

As mentioned above, the materials currently used to modify PLGA-based scaffolds can enhance the hydrophilicity of PLGA, improve cell adhesion, promote cell proliferation, and regulate cell function and differentiation. However, current research no longer focuses on applying a single material to scaffolds, rather a variety of materials are used, and the combined application of modified materials can endow these PLGA-based biological scaffolds with enhanced capabilities (Table 2). Large functional groups on the PDA surface not only improve the hydrophilicity and cell adhesion of PLGA-based scaffolds but they also bind other materials such as proteins [79,80,84,181], metal particles [77,182], exosomes [3,183], chitosan [184], hydroxyapatite [185], and drugs [185]. In order to regulate cell function, PDA surface adhesion proteins can enhance cell adhesion [181] and achieve a slow-release effect [79]. In addition, it has been reported that PDA possesses antibacterial properties and excellent photothermal agents and antioxidants; therefore, it can broadly be used as a modified PLGA-based coating [186]. Furthermore, shortcomings can be remedied by combining and applying improved materials such as hydroxyapatite, Ppy, bioactive glass, magnetic nanoparticles, and graphene. However, the inherent cytotoxicity drawbacks of polylysine and PEI have limited their use in cell therapy and tissue regeneration, in terms of their use as modified materials that coat PLGA-based scaffolds.

## 5. Conclusions and Future Prospects

PLGA is the most commonly used synthetic material for preparing fibrous scaffolds in tissue engineering, and it has been approved for clinical application by the US Food and Drug Administration (FDA) due to its biocompatibility and safety. Tissue injury due to external damage or diseases is an urgent problem. Cell loss in injured tissues results from cell death caused by ischemia or ischemia reperfusion. Replacing lost cells is the best way to fundamentally repair tissue damage; however, the survival and retention of directly transplanted cells at the injury site are extremely low due to the lack of oxygen and nutrient supply, reactive oxygen species, and the immune system’s microenvironment. Biocompatible biological scaffolds can act as modules to fill the lost tissue and provide a beneficial environment for cell growth. PLGA has also been favored as a biological scaffold in the field of tissue regeneration. Moreover, porous bio-scaffolds are more similar to human tissues in terms of structure, meaning that they are more conducive to nutrient delivery and metabolic waste elimination during tissue repair and regeneration. Therefore, this is a suitable environment for cell proliferation, migration, and differentiation, which may thus accelerate tissue repair and regeneration.

However, the inherent hydrophobicity of PLGA and the electrically neutral environment of its surface are not conducive to cell adhesion and growth. Improving the hydrophobicity of PLGA and enhancing cell adhesion is an inevitable challenge in terms of its ability to act as a bio-scaffold. Materials currently used to enhance the cell adhesion of PLGA bio-scaffolds include PDA, polylysine, Ppy, PEI, proteins, cell membranes, superparamagnetic iron oxide nanoparticles, hydroxyapatite, bioactive glass, and graphene oxide. The modification of PLGA by these substances can be divided into three forms, as follows: PLGA bio-scaffold coating, PLGA hybrid scaffolds, and PLGA complex scaffold. The representatives of PLGA bio-scaffold coating are PDAs and bioproteins: the former can enhance the surface hydrophilicity of the PLGA scaffold to enhance the scaffold and cell tissue adhesion, whereas the latter can interact with cell surface proteins to enhance cell adhesion and regulate cell function. PDA’s antioxidant and antibacterial properties make it particularly useful as a PLGA scaffold coating, given that it endows it with more abilities, although few articles discuss its role in tissue regeneration.

Here, as a method of surface modification of a PLGA scaffold, PDA coating can enhance the cell adhesion and growth, and drug loading can be carried out while surface modification is conducted to improve the therapeutic effect for patients such as in transplantation and bone formation. However, the degradation and metabolism of PDA coating in vivo are very important, but the metabolic pathways are still unclear. Compared with other methods, biological protein coating has natural advantages, good safety, and is similar to the extracellular matrix environment, which can well increase the hydropathy of PLGA and enhance the adhesion of scaffolds to cells through RGD sequence. However, it has the problems of protein source and economic cost.

Although the application of a single coating in tissue regeneration was the main topic of discussion in this article, an increasing amount of research has focused on the use of multiple coatings, as this can provide scaffolds with multifaceted properties. For example, using a PDA coating, followed by a protein coating, can significantly increase the effectiveness of the protein coating, and the hydrophilicity and cell adhesion of a composite-coated scaffold are also significantly improved, as compared with a single PDA coating. Currently, most tissue regeneration materials are composite applications of multiple materials. The advantage of applying a composite is that the limitation of one material can be overcome, and it is possible to take advantage of multiple materials in order to facilitate tissue regeneration. For example, a composite multifunctional scaffold that is made by immobilizing a small molecule activator (LYN-1604 (LYN)) onto the surface of a PLGA porous scaffold may use PDA as a bridge, followed by the in situ co-precipitation of HA nanocrystals [185]. The dual effects of osteoclast inhibition and osteogenesis stimulation can be achieved by the continuous orderly release of LYN and Ca ions.

However, the long-term safety of composite scaffolds in vivo may be a potential issue. When selecting coating materials, the interactions within the tissue’s microenvironment and immune system is crucial, as is their cell adhesion and biocompatibility. Recent studies have revealed that immune responses play an important role in regulating tissue regeneration. Several articles have discussed the positive and negative aspects of immune regulation in tissue regeneration [187,188,189,190,191,192]. Depending on the stage of tissue repair, and the immune response at different sites, optimal coating materials are selected to achieve immune modulation, enhance vascular regeneration, improve repair efficiency, and reduce scar formation. Designing biomaterial systems to target the dynamic immune microenvironment and interact positively with cells may elicit a more effective biomaterial-mediated immune modulation [193]. Drug delivery using biomaterial scaffolds can help to actively heal and immunomodulate injured tissues [194], or else the physicochemical properties of the material itself would affect the proliferation of adherent cells, and the topography and micropatterning of the scaffold’s surface could also influence the behavior of adherent cells.

Moreover, the acidic environment generated by the degradation of PLGA-based cellular scaffolds in vivo may induce tissue inflammation. Porous bio-scaffolds are more similar to human tissues in terms of structure. During tissue repair and regeneration, this environment is more conducive to nutrient delivery and metabolic waste elimination, and thus it provides a suitable environment for cell proliferation, migration, and differentiation, and it accelerates tissue repair and regeneration. Moreover, PLGA porous scaffolds do not seem to accumulate acidic products during degradation, and there is no significant inflammatory tissue response.

The surface modification materials and methods for PLGA-based cell scaffolds have made significant progress, and they will help to promote the further development of cell therapies.

## Figures and Tables

**Table 2:** Advantages/disadvantages of modified materials and applications.

Material Type	Function	Applications	Advantages	Disadvantages	Modification Method	Ref.
PDA	Contains multiple functional groups to enhance the hydrophilicity of PLGA	Act as a coating to improve the adhesion of PLGA-based scaffolds to cells, the adsorption of other materials	Strong adhesion to various materials, proteins, and cells, hydrophilic, antioxidant, good biocompatibility, etc.	Unknown metabolic pathway in vivo	Physical hybrid coating using surface functional groups	[71]
Polylysine	Positive surface charge to enhance PLGA hydrophilicity	Increases cell adhesion as a coating	Enhancement of negatively charged proteins and cell adhesion	Cytotoxicity	Physical hybrid coating using electrostatic adsorption	[72]
Ppy	Excellent electrical conductivity	Promotes tissue repair as an electrically conductive material	Good biocompatibility, good electrical conductivity	Poor cell adhesion and difficult processing	Ultrasound-assisted deposition	[73]
PEI	Positive surface charge	Alters PLGA-based scaffold potential and improves PLGA cell adhesion	Enhanced cell adhesion	Cytotoxicity	Physical hybrid coating using electrostatic adsorption	[74,75]
HA	Improves hydrophilicity and enhances cell adhesion	Interacts with cell membrane receptors and regulates cell viability	Good biocompatibility can participate in cell signaling and wound healing, etc.	Poor mechanical strength and negative charge affect cell adhesion	Physical mixing followed by chemical cross-linking	[84]
Gelatin	Enhanced cell adhesion	There are RGD sequences that enhance cell adhesion and induce biochemical responses	Non-cytotoxic, RGD-rich sequences, good conductivity, etc.	Poor mechanical strength	Vacuum-assisted filling or chemical cross-linking	[181]
Type I collagen	Enhances cell adhesion and regulates cell function	Binds to cells and regulates cell function	Good biocompatibility, modulation of cell function, increased cell adhesion, etc.	Poor mechanical strength	Chemical cross-linking	[75]
Laminin	Enhances cell adhesion and promotes nerve regeneration	Interacts with multiple cellular receptors and signaling pathways and regulates multiple cellular activities	Cell adhesion factor ligands such as integrins are available to enhance cell adhesion, biocompatibility, and low immunogenicity	Poor mechanical properties	Chemical cross-linking	[76]
FN	Enhances cell adhesion	Interacts with multiple cellular receptors and signaling pathways and regulates multiple cellular activities	Enhanced cell adhesion through integrin pathway, broad functional activity, low immunogenicity, etc.	Poor mechanical properties	Chemical cross-linking	[77]
Cell membrane	Enhances cell adhesion and regulates cell function	Enhances cell adhesion and regulates cell function	Low immunogenicity, pluripotency	Poor mechanical properties	Chemical cross-linking, direct deposition	[78]
Filipin protein	Improves PLGA cell adhesion	Enhances cell adhesion and improve PLGA hydrophilicity	Good biocompatibility and biodegradability	Poor mechanical properties	Chemical cross-linking	[79]
Magnetic iron oxide nanoparticles	Empowering PLGA with special functions	Promotes stem cell differentiation, antibacterial, promote osteoblast proliferation, etc.	Multifunctional, metabolizable, malleable, etc.	Cytotoxicity	Layer-by-layer assembly	[80]
Hydroxyapatite	Increase cell adhesion	Nanosize enhances PLGA adhesion to cells	Good biocompatibility, osteoconductivity, bioactivity, etc.	Mainly used for bone repair	Ultrasound-assisted coating, sputtering deposition, layer assembly, or physical hybrid spontaneous deposition	[81]
Bioactive glass	Increase cell adhesion	Increases adhesion to cells and induces bone formation	Good biocompatibility, bone conductivity, ability to bond with living bone, etc.	Low brittleness and toughness	Solvent casting particle leaching method	[82]

## Data Availability

Not applicable.
